# The Topology of a Discussion: The #Occupy Case

**DOI:** 10.1371/journal.pone.0137191

**Published:** 2015-09-09

**Authors:** Floriana Gargiulo, Jacopo Bindi, Andrea Apolloni

**Affiliations:** 1 NaxYs, University of Namur, Namur, Belgium; 2 DISAT and Center for computational Sciences, Politecnico di Torino, Torino, Italy; 3 Department of Infectious Disease Epidemiology, London School of Hygiene and Tropical Medicine, London, United Kingdom; Universiteit Gent, BELGIUM

## Abstract

**Introduction:**

We analyse a large sample of the Twitter activity that developed around the social movement 'Occupy Wall Street', to study the complex interactions between the human communication activity and the semantic content of a debate.

**Methods:**

We use a network approach based on the analysis of the bipartite graph @Users-#Hashtags and of its projections: the 'semantic network', whose nodes are hashtags, and the 'users interest network', whose nodes are users. In the first instance, we find out that discussion topics (#hashtags) present a high structural heterogeneity, with a relevant role played by the semantic hubs that are responsible to guarantee the continuity of the debate. In the users’ case, the self-organisation process of users’ activity, leads to the emergence of two classes of communicators: the 'professionals' and the 'amateurs'.

**Results:**

Both the networks present a strong community structure, based on the differentiation of the semantic topics, and a high level of structural robustness when certain sets of topics are censored and/or accounts are removed.

**Conclusions:**

By analysing the characteristics of the dynamical networks we can distinguish three phases of the discussion about the movement. Each phase corresponds to a specific moment of the movement: from declaration of intent, organisation and development and the final phase of political reactions. Each phase is characterised by the presence of prototypical #hashtags in the discussion.

## Introduction

The number of Twitter users has increased hugely in the past few years, and today Twitter has almost half billion users worldwide (http://semiocast.com/).

Twitter is a micro-blogging platform, where users can share 140 character messages, called tweets. Users are identified by the @ symbol proceeding their unique identifier (twitter handle), while topics of the tweets are identified by the symbol # followed by a single-word phrase. These semantic elements are called hashtags and allow the direct search of the tweets concerning a certain topic. This means that the information flows are not obliged to pass through the social network structures. Therefore, since tweets are in principle visible by all the users, Twitter is meant more as an information sharing service than a social network facilitator.

To increase the visibility of a tweet, users can re-tweet them or introduce hashtags in the tweets and engage in conversations with other users, who couldn’t be directly reached.

Almost 340 million tweets (https://blog.twitter.com/) are sent every day and can be collected using a freely available application programming interface (API). In this way, Twitter represents a goldmine of data in a variety of fields including analysis of social network communities [1], influenza detection [1–3], political topics [4][5], sentiments about childhood vaccination [6] and predictability of social events [7]. The number of studies based on the analysis of tweets is so big that some scientists have pointed out the importance of ethical guidelines for this type of research, as in the case of animal or human involvement [8].

Twitter has been recognised as playing an important role for the organisation and communication of the recent civil uprisings in North Africa (the Arab Spring) [9], the London riots in 2011 [10], the Hong Kong Occupation and the Occupy Wall Street (OWS) movement [11,12]. Previous works studying the relation between social movements and the media have focused on describing how Twitter has helped the spread of information, the users’ characteristics and their geographical distribution. In particular the works by Conover and collaborators [11,12] are directly related to the OWS movement. The authors [11] have studied communication flows among Twitter users in the same state and across borders. They found that at the domestic level communication focuses on protest activities (recruiting activity); at the inter-state level there are references to the media (propaganda activity). The authors [12] analysed the users’ activity and the evolution of their engagement in the movement (from recruitment to advocacy and loss of interest); the most active ones shared pre-Occupy interests and whilst highly vocal at the beginning, lost interest eventually. Similar analyses exist for the 15 May movement (from now on 15M) in Spain [13,14]. The authors have re-constructed the social network among users through their re-tweeting activity. The network has been analyzed to study structural characteristics, the presence of communities and the connection between online networks, social contagion and collective dynamics.

The aim of our paper is to use Twitter data to study how the political discussion evolved during and after the OWS movement—identifying how the users' activities and discussion topics have co-evolved during the time.

We concentrate our attention on a particular graph structure that is the bipartite network between users and hashtags: this helps to capture the relationships between actors and contents of the debate. We use social network tools to identify the role of particular users and topics in the spread of information and in the evolution of the information content.

The abovementioned works [11–14] differ from ours mainly in two aspects. First of all with respect to the way data have been collected and consequently how social networks have been re-constructed: networks have been re-constructed starting from the re-tweeting (direct relation) while in our case the network has been built using a bipartite approach (see Data collection and Network setup) thus emphasising the common interests among users (indirect relation). Secondly, and more importantly, all these works focus on the user activity and engagement, however little information can be extracted about how the discussion has evolved during the period of the movement. This probe has an important insight on the phases of the movement (from birth to engagement to dissolution) and narrates the movement.

The work is organised in this way: in section Data collection and Network setup we provide some information about the social movement and the data mining procedure. We also show how the collected anonymised data have been used to construct the two networks (one among users and the other among hashtags) that we will use for the analysis; in section Static network results we present the results of the network topological analysis for the two mono-partite projections. We also show the community structure in the semantic network. In section Temporal features we show the results on the communities’ evolution and the network during the movement. Finally in the Conclusions we detail further perspectives of this work.

## Data Collection and Network Setup

On the 13^th^ July 2011, the counter-cultural magazine Adbusters publicized a call to occupy Wall Street financial district on the September 17^th^ (https://www.adbusters.org/). Following the call to action, on that day hundreds of people gathered at Zuccotti Park. This date marked the birth of the 'Occupy Wall Street' movement, from that day onwards more than 200K persons got involved. The Occupy movement organised massive demonstrations until May 2012. In a few weeks the practice of occupying public spaces spread to over 80 cities in the USA along with the name 'Occupy'. Occupy activists used Twitter to organise operations on the ground, to discuss or share news and ideas. Hashtags as '#Occupy', '#ows', '#occupyoakland' played a key role in Occupy’s communication and gained global exposure on Twitter.

While previous papers mostly studied the protesters’ social network structures, we focus on the relations between the users' behaviour and the semantic content of the discussion. For this reason our dataset is a random sample of the Twitter messages including the hashtag ‘#occupy’ or the word ‘occupy’ in the text. We chose to avoid a snowballing procedure in the mining phase, clearly more suitable for using the re-tweeting approach for building the users network. Instead, using a random sampling, we emphasize the Twitter’s characteristic of giving a direct access to the hashtags bypassing social relationships. Data have been collected using the standard Twitter Search API and therefore are limited to a maximum of 1500 tweets per day that can be downloaded.

The dataset includes 180K short messages, collected between the 26^th^ October 2011 and the 18^th^ April 2012, written by more than 37K different users and contains 17K hashtags. For some days there were problems in collecting the tweets through the use of the Twitter Search API. The anonymous data can be found in the S1 Data file.

In the Supporting Information (S1 File) we show some comparison with the Spanish #15M movement using the same freely available (http://cosnet.bifi.es/wp-content/uploads/2013/03/) dataset described in [13–15]. This dataset has a smaller time range than ours, but larger number of tweets per day.

**Fig 1:**
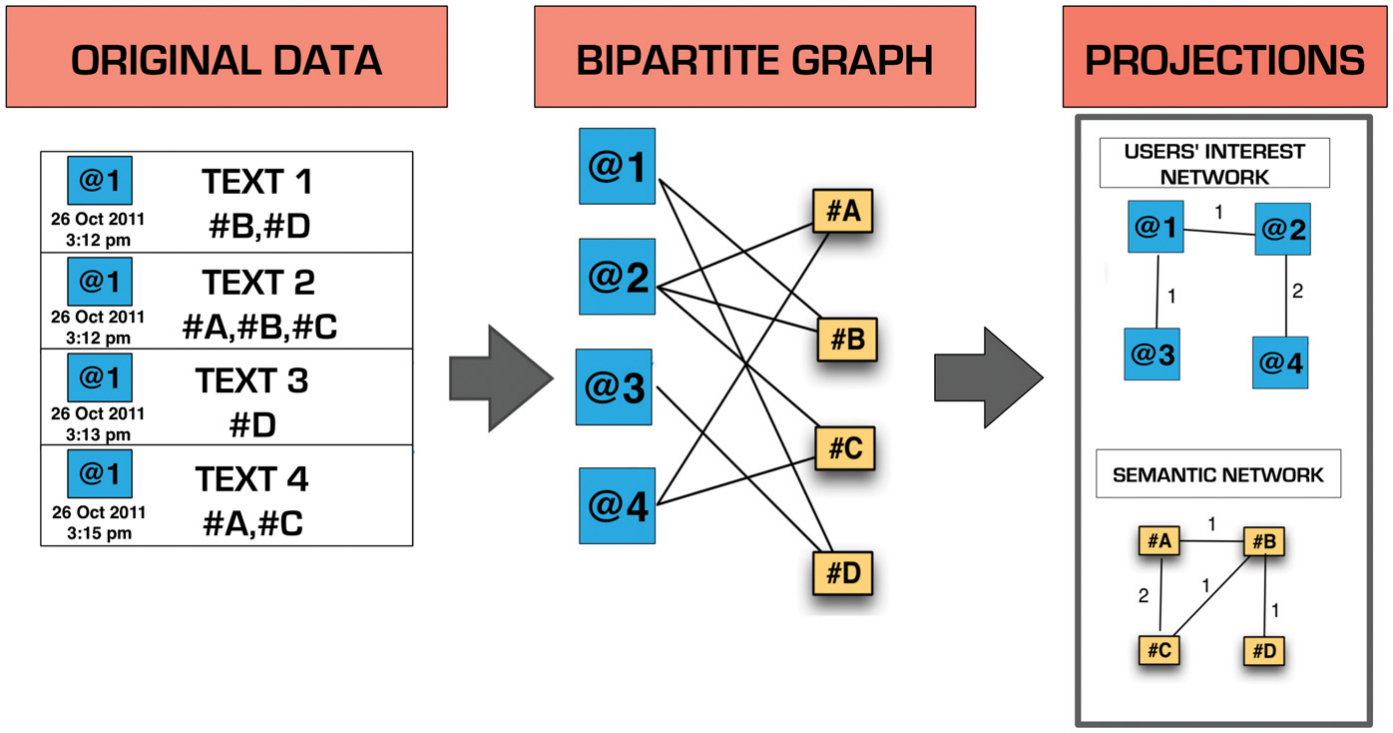
Schematic representation of the bipartite graph construction from raw data and of the projections on the semantic and interest mono-partite graphs.

To study the interaction between the users’ activity and the semantic contents, we used a method similar to the one used by Roth [16] for analysing the blogosphere. Firstly our raw data have been organised to form a bipartite graph @Users-#Hashtags (Fig 1) using standard techniques [17–19]. From this, we can define two mono-partite network projections: the 'semantic network' for the #hashtags and the ' users’ interest network' for the @users. In the semantic network, nodes correspond to #hashtags and a link (undirected) between two nodes exists if at least a @user uses those two #hashtags. The weight of the link is given by the number of @users using the same pair of #hashtags. Similarly, in the users’ interest network, two nodes (@users) are connected if they use the same #hashtag and the weight of the link is given by the number of #hashtags that are shared.

We consider both the static, temporally aggregated, and dynamical network, described by its daily snapshots.

The aggregate network contains a much larger amount of information than when its daily counterparts are considered separately. In fact in the former case, the network includes all the links among nodes (#hashtags or @users) that have been created on different days. Therefore, while the daily structures are necessary to evaluate the dynamics of the debate, the static network is the necessary tool to understand the discussion’s semantic connections and its evolution.

Given the query mechanism, the hashtag #Occupy appears in most tweets, giving origin to an almost fully connected network. To better describe the role of other hashtags in the communication flow and in the patterns’ formation, we have removed the #Occupy hashtag in the construction of the users’ interest network.

## Static Network Results

In this section we show how basic network analysis tools, applied to these particular network structures, can provide important information on the semantic level and on users’ participation in the movement debate.

### The semantic network

The semantic network is formed by 5206 hashtags, connected by 95543 links.

**Fig 2:**
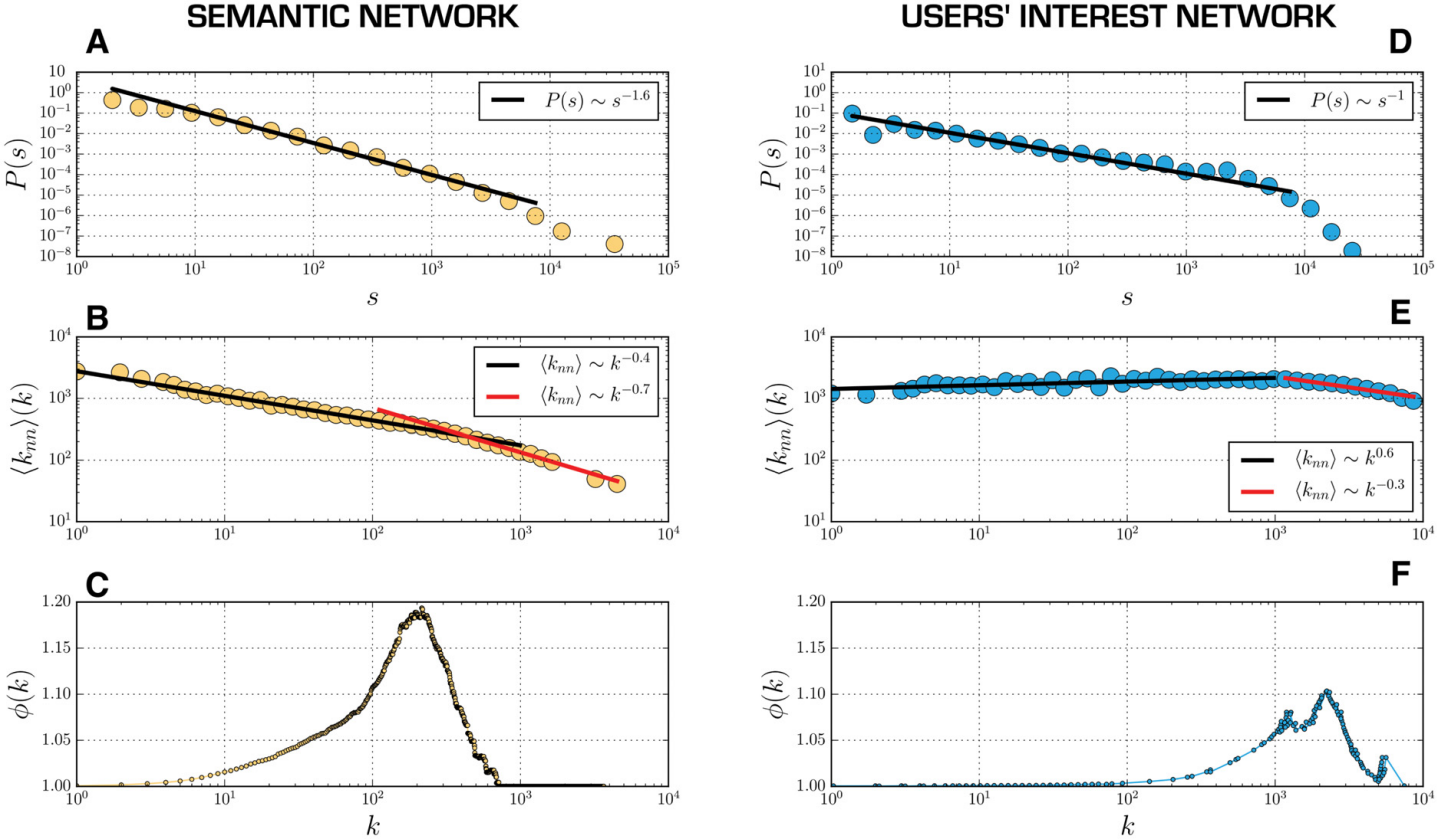
Stength, degree correlation and rich club index distribution for the two network projections. Plot A and D: Strength distribution of the semantic (A) and users’ interest network (D). The distributions follow a power law with exponents respectively *γ* = -1.6 and *γ* = -1 respectively. In the case of the users’ interest graph a pronounced exponential tail is observed. Plot B and E: Degree correlation of the semantic (B) and users’ interest network (E). For the semantic graph the average neighbours' degree decreases sub-linearly with the degree. The decrease is faster for hubs. For the users’ graph the average neighbors’ degree increases very slowly with the degree for *k*<10^3^. For the hubs (*k*>10^3^) we observe a sub-linear decrease. Plot C and F: Normalized rich club index as function of degree for the semantic (C) and the users’ interest network (F) For both networks the index is increasing with the degree indicating the existence of correlations in the tow networks. The decrease for large value is an effect of the networks finite sizes.

This network presents a scale free network structure. As shown in Fig 2A the nodes' strength distribution (i.e. the sum of the links’ weight originating at the node) follows an evident power law (the same can be observed in degree and weight distributions in Figure A in S1 File) few semantic hubs are present, through which most of the ''semantic traffic" is channelled through.

The presence of semantic hubs has been observed in several large-scale semantic networks as an effect of the preferential spreading of the words in different contexts (the most important words are more likely to acquire new connotations)[20]. In our case the most important hashtags are more likely to be chosen by the users (also due to the Twitter’s “trending topic” ranking system which displays the most popular hashtags). In this sense, the debate around the social movement develops around these hubs that can be identified as the pivotal points of the discussion.

To study the network's mixing properties (Fig 2B), we use the average degree of the neighbours,

$$\langle k_{nn}\rangle \;=\;\frac{\left({\sum^{\text{​}}}^{\text{​}}{}_{j\in V_{i}}k_{j}\right)}{k_{i}},$$

where the sum is extended over all the nodes directly connected to the i-th node (*V*_*i*_). This quantity decreases with *k*, denoting a preference for low degree nodes to be connected to high ones. This confirms the idea of a debate that gradually grows, creating local connections around the hubs.

To gain some insights about possible correlations in the network and the role of hubs we use the rich club index (*ρ*(*k*)). This index measures the fraction of edges (*E*_>*k*_) that are actually connecting nodes with degree larger than *k* (*N*_>*k*_), over the maximal number ($\frac{N_{>k}(N_{>k}-1)}{2}$) of edges between these nodes as in [21]

$$\rho (k)\;=\;\frac{2\;E_{>k}}{N_{>k}(N_{>k}-1)}$$

A large value of the index indicates that hubs are more likely to interact among themselves than with others. To avoid problems related to the final size of the network a better measure is the normalised rich-club index (*ϕ*(*k*)): *ρ*(*k*) is normalised by the rich club index of a random network with the same degree distribution $\rho_{rand}(k)$

$$\phi \left(k\right)\;=\;\frac{\rho \left(k\right)}{\rho_{rand}\left(k\right)}.$$

In Fig 2C we show the behaviour of the normalised rich club index. A value of *ϕ*(*k*)>1 indicates the presence of a rich-club effect leading to the formation of a oligarchy of strongly communicating hubs that provide the backbone for the information flow. Here we observe that the rich club effect is present for a large class of high-degree nodes but not for the largest hubs. This identifies a hierarchical structure of the nodes: large hubs are rarely connected to themselves and are connected to the majority of the lower-degree nodes; smaller hubs are strongly connected to themselves and constitute the backbone of the debate; peripheral nodes that are mostly linked only to important hubs.

### The semantic clustering

**Fig 3:**
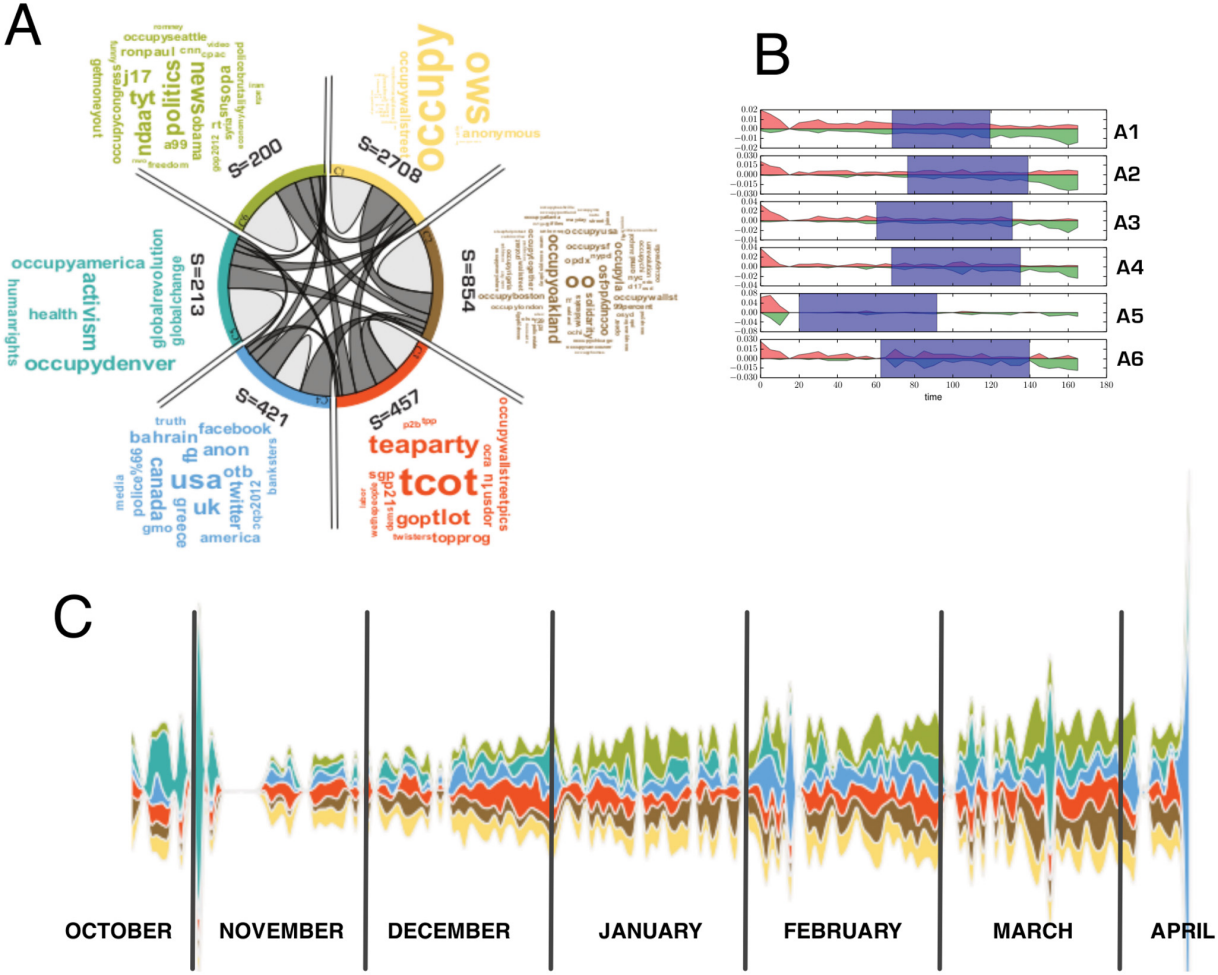
Communities description of the semantic network. The semantic network can be divided in 6 communities. Plot A: chord diagram representing the interactions among communities and their strengths. The grey bands represent links among communities member and their width is proportional to the number of connections between the two. Plot B: the permanence time of hashtags in the communities. The red histograms indicate the fraction of hashtags entering the community, for the first time, at a specific time; the green one the leaving time. The shaded area corresponds to the average permanence time of the community contents. Plot C: steamgraph showing the relative activity of the communitiesat time t. The relative importance of the community at time t is evaluated as the number of tweets contacting at least one of the community ahshtags normalized tot toal number of tweets collectd at time t. For each day, the height of the band corresponds to the proportion of activity of each community (number of tweets) normalized to the total activity. The color code is the same as in plot A and each band in the stream graph corresponds to a specific community (C1 in the bottom,C6 on top).

We analysed the community structures of the semantic network, using the Louvain community detection method [22]. The semantic network is composed of six macroscopic communities with a clear social interpretation (Fig 3).

The network hubs are equally distributed among the communities and perform the connections among the different topical areas as shown in Fig 3A. The activity patterns of the six communities are shown in Fig 3C, describing the relative importance of each in the different phases of the movement’s evolution.

We define the entry time into the discussion as the first appearance of the hashtag ($t_{i}^{MIN}$), the exit time as the time of the last appearance ($t_{i}^{MAX}$) and the permanence time as the interval between the two.

We define the time span of a community (T_I_) as the average of the permanence times of its members

$$T_{I}\;=\;\frac{\left(\sum_{i\in I}t_{i}^{MAX}\right)}{N}-\frac{\left(\sum_{i\in I}t_{i}^{MIN}\right)}{N}$$

where *N* is the number of community members.

In general, most of the hashtags are introduced at the very beginning of the discussion and exit at the end. In fact, most of the communities have similar initial and final times, centered on the central day of the data collection. This is true for all the communities except for community C5—characterised by the use of very early created hashtags (Fig 3B). It is important to note that the first days of the data collection correspond to the phase when the movement was occupying Zuccotti Park (17^th^ September to 15^th^ November 2011) as shown in Fig 3C. Therefore the C5 community, contains hashtags relative to the important themes discussed during the assemblies in Zuccotti Park (e.g. #poverty, #liberty, #humanrights) and defines some of the values that have characterised the movement. After the 15^th^ November the movement continues its discussions mostly on social media platforms, specifying its important topical hashtags. The contents of community C5 are mostly shared with the first (C1) and the second (C2) communities. Community C1 (the largest) contains all the important keywords connected to the different typologies of globally-spread activism from this kind of social movement (e.g. #ows, #tharir, #anonymous, #revolution). Community C2 contains several local denominations of the Occupy movement (e.g. #oakland, #cal, #philly) and significant dates (e.g. #j20,#j28,#j29, #d7 but also #mayday, #1m). This is, therefore, a community mostly addressed to the organisation of the events. Community C3 is mostly focused on discussions around the elections and institutional politics (e.g. #teaparty, #tcot,#tlot). Community C4 concerns the internationalisation of the occupy movement (e.g. #eu, #greece) and several movement keywords that become international (e.g. #99%, #banksters). The last community C6, for which a high number of tweets were introduced after the eviction of Zuccotti Park, mostly contains discussions about the repression of the movement (e.g. #policebrutality, #pepperspray).

### The users’ interest network

The users’ interest network is formed of 12485 users connected by 3962037 links.

The users' interest network is also characterised by a power law behaviour for the strength, the degree and the weight distributions (Figure B and S1 File). Both the degree and strength distributions have an evident exponential cutoff at the tail of the distribution.

Following the procedure in [23] we distinguish two classes of communicators based on their topological properties: the 'professionals' and the 'amateurs'. Professional communicators (like bloggers, journalists, official media representatives) and spybots are concentrated in the tail of the distribution. In this sense the origin of the exponential tail (phenomenon that is usually attributed to the finite size of the system) can be attributed to the different activities’ patterns of the prototypical users’ profiles: ‘amateurs’ use social media less intensively, selecting the hashtags according to their preferences; ‘professionals’ and ‘bots’ are more active and use all the semantic space until saturation (See Figure C in S1 File).

Due to the heterogeneity of the network, the largest part of the professionals’ connections are towards less connected nodes (Fig 2E). This feature is typical, for example, of communication networks, where the structure is meant to maximise the spread of communication [24]. Whereas ‘amateurs’, or occasional users, have few connections, indicating a preferential choice for semantic contexts and interact mostly among themselves. This creates the complex mixing pattern that can be observed in Fig 2E.

Professionals are largely connected among themselves (since they tweet all the important hashtags to gain visibility). This is demonstrated by the normalized rich club index (Fig 2F) and this assumption is reinforced by the analysis shown in S1 File.

### Network robustness

An important feature for social movements is to understand if, and under which condition, the use of Twitter as a communication platform, is robust to repression measures. As a measure for the robustness we use the standard one presented in [25] that implies the use of a dynamical failure model: robustness is evaluated as the largest portion of the graph that remains connected after a certain fraction of nodes are removed based on their degree (targeted attack). In our case, the sequential removal of nodes (hashtags) in the semantic network mimics censorship measures, while in the users' network it represents the accounts' removal.

To understand the connection between the robustness of these networks and their structural properties, both for the semantic and for the users’ interest graphs, we compare the robustness evaluation procedure with the same procedure on two different reshuffled graphs: the first reshuffling method ("*ab-initio* method") randomizes the position of the existing hashtags in all the messages, namely, the hashtags are randomly distributed among users. This method allows eliminating all the semantic preferences of the users before the network reconstruction. The second reshuffling method ("topological method") consists in the construction of graphs with the same degree sequences (global properties) as used in the configuration model [26]. The reshuffled networks obtained with this method loose all the local properties of the original network, like the assortativity and the clustering. Therefore, using this method as benchmark allows to understand the role of the local topological properties for the robustness.

**Fig 4:**
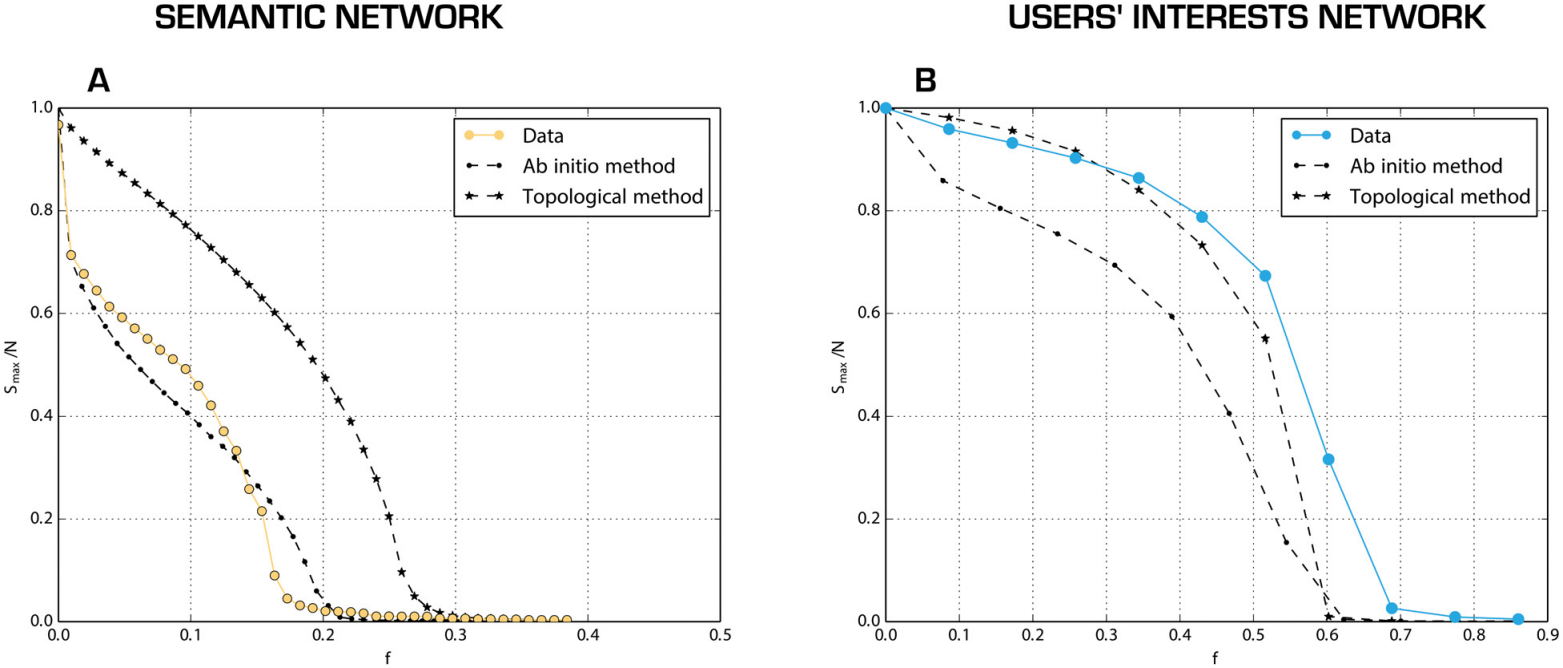
Robustness of the two mono projections of the network as a function of the removal of nodes (target strategy). On the y-axes the size of the largest connected part of the network normalized to the size of the network, on the x-axis the fraction of nodes removed. On the left semantic network, on the right users interest one. The colored lines correspond to the results relative to the original networks. The black lines correspond to cases when network has been re-shuffled using *ab-initio* (dashed line) and the topological method (star-lined). The results are averaged on 100 replicas of the attack model and, for the rewired networks, for 50 replicas of the reshuffling process.

The semantic network is less robust than its counterparts after topological reshuffling as seen in Fig 4A. This is due to the strong hierarchical structure of the network since most of the connections are toward hubs. The topological reshuffling reduces the disassortative behaviour of the network, increasing its robustness. The *ab-initio* reshuffling for the semantic networks increases the number of links between low degree nodes, thus still ensuring connection among them, making the network more resistant to targeted attacks.

As we can observe in Fig 4B, the users' network is more robust to users' deletion than its counterparts after topological reshuffling: that is, a large number of users should be censored, before impeding the flow of information. This effect is mainly due to the high connectivity among amateurs. These users are the core of the activists who are deeply involved in the discussions about the movement: they share among themselves only the very important keywords for the movement, and filter out other information. Their strong connectivity guarantees the network a high level of robustness. The users' interest graph is more robust respect to its *ab-initio* reshuffled copies due to the low value of the weights (because of the differentiation of users' semantic preferences): the *ab-initio* reshuffling creates in this case less connections with stronger ties.

We can conclude that Twitter is a robust communication tool. The robustness can be improved creating more links among less popular hashtags (low degree nodes in the semantic network). From a user point of view, this can be achieved through adding new or uncommon hashtags into the tweets, together with the most common hashtags. In this way, also in the case of censorship of the most common hashtags, the information can still flow through less common ones.

## Temporal Features

We reconstruct the temporal daily networks G(V,E,t), both for the semantic structures and for the users. We studied how semantic and participatory innovation is present in the network evolution. To this end, we analyse the turning points in the dynamical networks’ evolution proposing a statistical procedure similar to the one presented in [27] founded on time series analysis.

**Fig 5:**
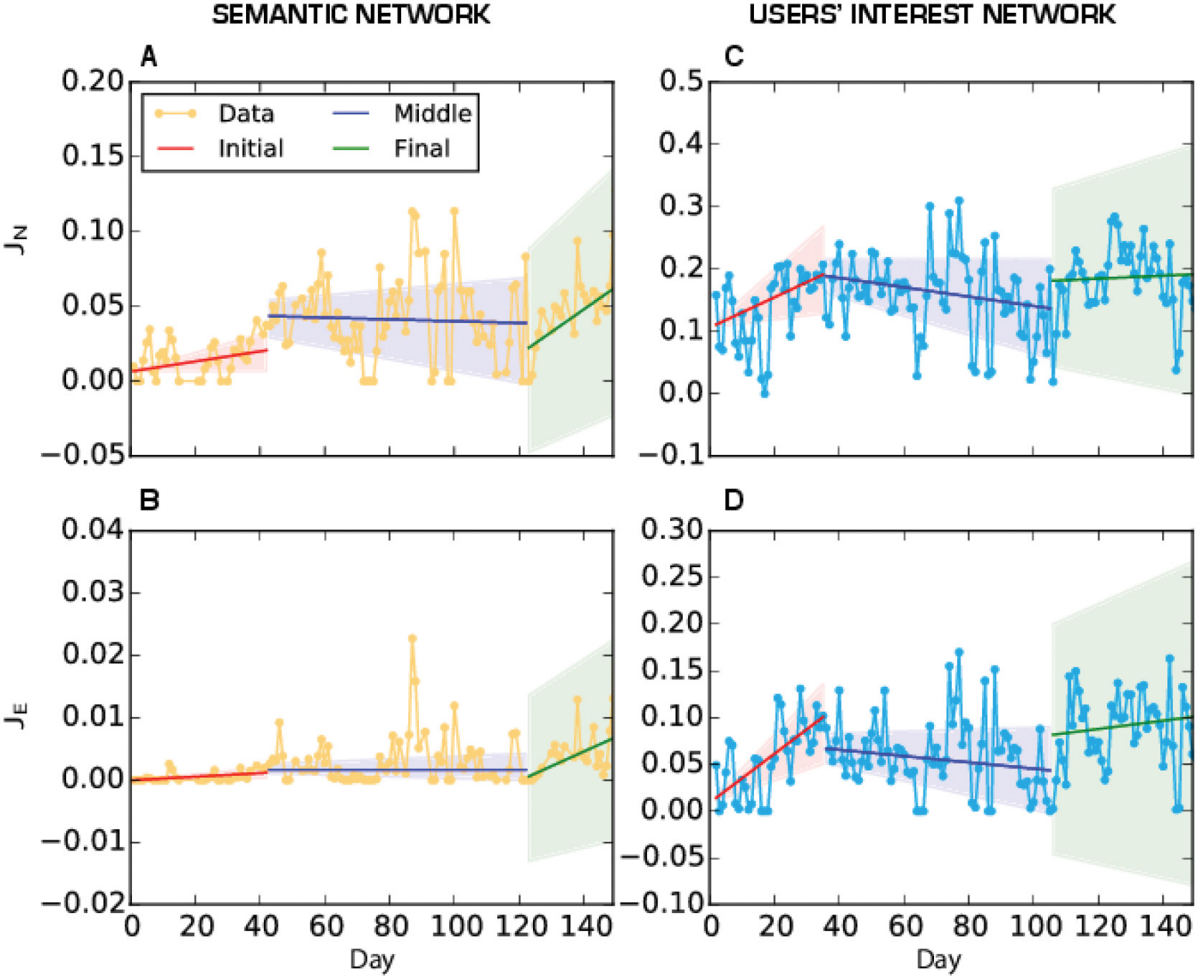
Time series for the Jaccard index for the two networks: semantic on the left, users’ interest on the right. For each of the three phases(Initial, Middle and Final) we have estimated the interpolating lines using Theil-Sen regression and the shaded area correspond on the 95% Confidence Interval. Figures A and C: dots represent the values of the Jaccard index for the Nodes at time **t** and at time **t-1**. Figure B and D: dots and line represent the values of the Jaccard index between the set of edges of the at time **t** and at time **t-1**.

At each time step *t*, we evaluate the difference between the network at time *t* and *t*-1. The difference is evaluated considering the Jaccard index between the set of nodes at *t* and at *t*-1 (*J*_*N*_(*t*)) and between the set of edges at *t* and at *t*-1 (*J*_*E*_(*t*)). The Jaccard index is a measure of similarity between two sets A and B and is determined by the ratio between the size of the intersection (common elements) and the union (total elements) of the two sets. In our case the two sets correspond to the set of nodes and edges of two different temporal snapshots of the network. A low value for the index indicates that the two networks are very dissimilar and that in between two measurements an innovation has been introduced; vice versa, a high value of the index indicates continuity in the process. After reconstructing the time series of the Jaccard indices (for *J*_*N*_ and *J*_*E*_) we evaluate the trends in the temporal series (Fig 5) using the Mann-Kendall test [28]. The test shows that for the time series of two Jaccard indices, trends exist for both networks and are significant at the level of 5%.

However the trend is not constant and we have identified three prototypical phases in the semantic evolution of the debate: Initial (days 1–40), Middle (days 41–120) and Final (days 121–175). In these three phases the trends vary as can be seen from the results of Mann-Kendall tests in Fig 5. In Fig 5 we show the fitting lines for the different phases using three different colours. We can see that during the first phase of the discussion, coincident with the period of the occupation of Zuccotti Park, the innovation level is very high (very low Jaccard index) both in the topics (semantic network) and in the participation (users' network). At the beginning, the Jaccard index follows a positive increasing trend for both networks. After the eviction of Zuccotti Park, the Jaccard index shows a decreasing trend in the semantic network while there is no trend in the users’ interest network. This indicates a phase of consolidation of the movement where users strengthen their relation with others using pre-existing hashatags. In the last phase the Jaccard index increases again for the users’ network indicating that new users are appearing. For the semantic network, there is no apparent trend but, as we know from the community analysis (see below) the new hashtags created are not directly inherent to the movement. This getting out of the original context favors the entering of new users not directly interested in the occupy movement.

**Fig 6:**
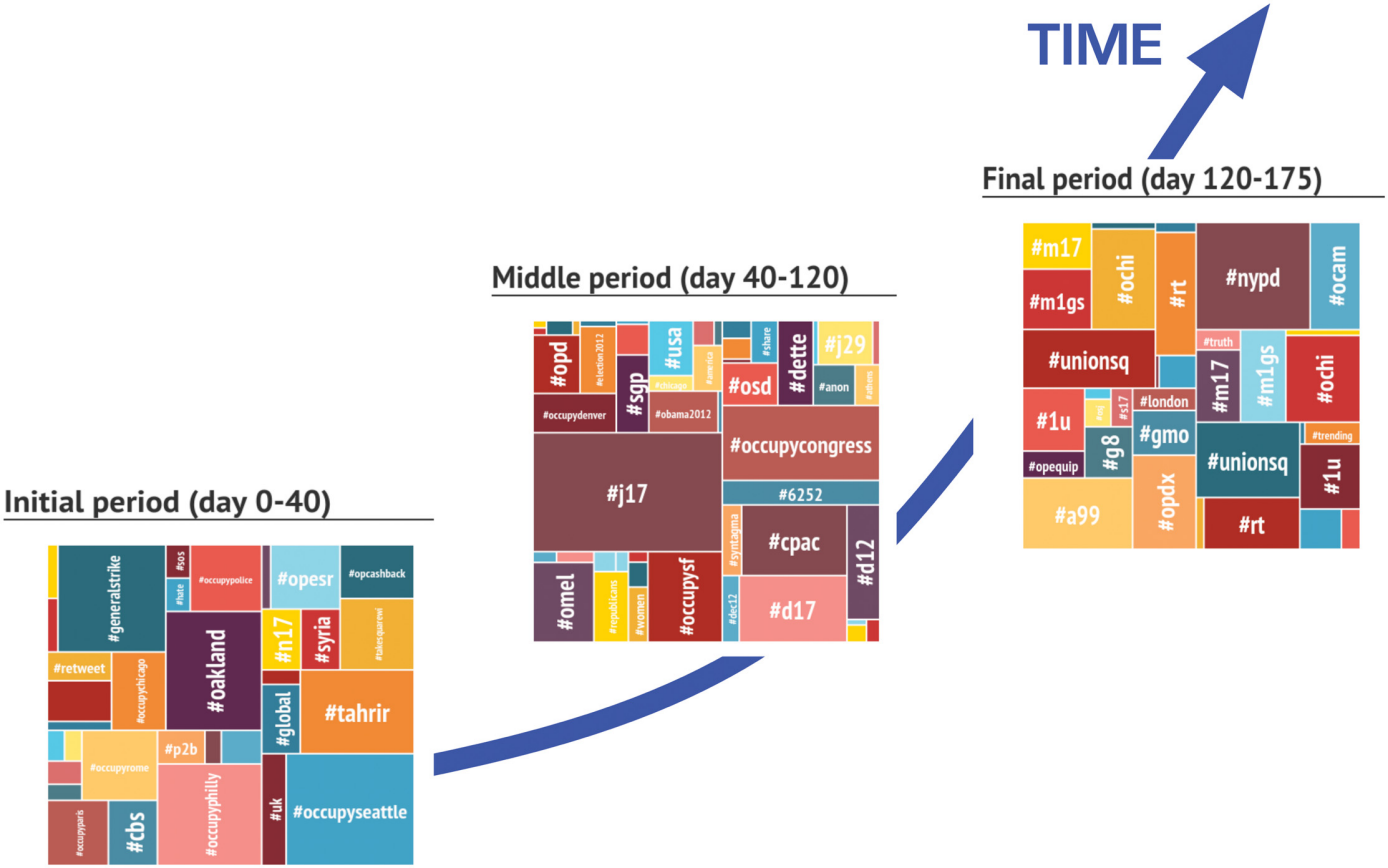
Top hashtags in the three phases of the Occupy Wall Street Movement. Each square corresponds to one specific period of movement and report the top most used hashtags in the period. The size of the square around each hashtag is proportional to the number of times it has been used.

Using this division into three important debate eras, in Fig 6 we show hashtags appearing in the initial phase of the movement (days 1–40, around Zuccotti Park occupation) and after disappearing, the ones appearing for the first time in the middle period (days 41–120) and then disappearing, and finally the ones emerging in the last phase (days 121–175) of the data collection. We notice that the hashtags strictly relative to the first phase were mostly linked to the Occupy movement activism (e.g.#takethesquare, #generalstrike,#opcashback,#opESR) and to local declinations of the movement that started simultaneously worldwide (e.g. #occupyphilly, #occupyseattle, #occupyrome, #occupyparis).

In the middle phase the discussion is enriched with several keywords concerning the institutional politics (e.g. #elections2012,#obama,#republicans) and with the scheduling of demonstrations (e.g.#j17) [29]. Finally, the latest phase is characterised by several keywords concerning the political repression of the movement and more general societal topics (notice that hashtags containing the substring “occupy”, never occur, for the first time, in this phase).

**Fig 7:**
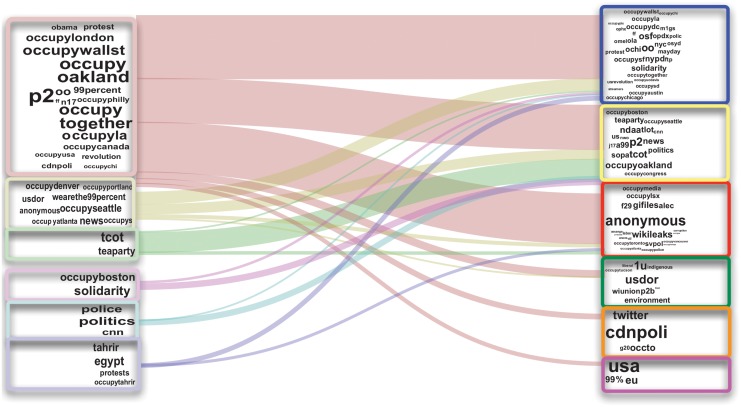
Evolution of the users’ participation to the semantic communities during Occupy Wall Street Movement. On the left end we have the hashtags used in the communities in the first phase of the movement; on the right end we have the same at the end of the movement. Connecting lines represent the flows of users whose interests in the discussion have changed during the period of the movement as represented from the different types of hashtags they use (i.e. users using hashtags in community *i* at the beginning and in community *j* at the end).

As the discussion is going on, new topics arise and old ones become less popular (or change their semantic connotation) and thus users change their interests. Also the semantic topological communities evolve, with merging and splitting processes [30,31]. Users’ interests coevolve dynamically with the semantic clustering. As users’ interests evolve, we can observe a flux of users from one semantic community to another. In Fig 7 the alluvial plot shows the flow of users among the different topics during the period under observation: the word cloud represents topics in the communities at the beginning and at the end; bands represent the flows of users from one topic to another, that is the number of users whose interests in the discussion have changed during the period. As time passes, discussion becomes more and more focused on three major theme areas. Less popular topics disappear and users originally using these hashtags move to more mainstream topics. Furthermore, new topics emerge giving rise to the creation of new small communities at the end of the process.

## Conclusions

In this article we have analysed a set of tweets relative to the movement ‘Occupy Wall Street’ with the aim of studying the semantic evolution of the debate through the users’ activity. Using standard network tools we have built a bipartite network whose nodes are @users and #hashtags extracted from Twitter. We have studied the two projections: the semantic network between the hashtags and the users’ interest network. We show that the topological structure of the network can help to identify the roles of different actors of the debate: amateurs and professionals. Also the hashtags have different connectivity properties according to their importance, as seen in the degree mixing and the rich club effect. We studied the robustness of the network showing that both projections are resilient with respect to deletion of nodes: this means that, under a regime of censorship, messages could still spread among activists.

Using community detection algorithms we have found different semantic communities that indicates topics that are strongly related among them. We noticed that as the movement has evolved, the discussion has moved from declaration of intent to media and institutional reactions. The use of the Jaccard index time series has allowed us to establish three different phases of the discussion evolution based only on the characteristics of the network. As the debate evolves in time, users are more likely to change interests and this is reflected by the use of hashtags in tweets. As we see, in the end the discussion is focused on three main topic areas.

## Supporting Information

S1 Data Zip file containing the anonymous dataset that can be downloaded from the Dryad Digital Repository: http://dx.doi.org/10.5061/dryad.q1h04. (ZIP)

S1 File File containing additional analyses not presented in the main file for sake of clarity. (DOCX)
