# Supplementary material for: The Topology of a Discussion: The #Occupy Case
Source: PLoS One. 2015 Sep 9;10(9):e0137191. doi: 10.1371/journal.pone.0137191 (PMC4564107; doi:10.1371/journal.pone.0137191)
Supplement: S1 File — (DOCX) [file pone.0137191.s002.docx]

Additional topological features

# Semantic network

In Figure A A,B we show that the degree and the weight distributions of the semantic network follow a power law too.

Further analysis suggests that hubs' local topology is different from the low degree nodes. For example, network hubs exhibit a stronger preference for connecting to low degree nodes as seen in the main text. The strength of the nodes, i.e. the sum of the links weights originating at the node, scales sub-linearly with the degree (Figure A C). This effect is mostly due to the weights heterogeneity and to the fact that for most of the links $w=1$ (i.e. only a user is putting these two hashtags in the same tweet). Since strong ties are equally shared among the hubs, the low weights of the connections between hubs and low degree nodes, gives origin to the stronger sub-linearity of the hubs behavior.

Because of this, a clear hierarchy in the semantic network is defined: all the heaviest links connect network hubs that, in turn, have strong ties only with other hubs. A numerical proof of the prevalence of strong ties among hubs is given in Figure A D showing that the weight is independent from the product of the nodes degrees for low degrees nodes, whilst grows extremely fast with the product of the nodes degrees for hubs.

Figure A. Plot A: Degree distribution of the network. The distribution follows a power law behaviour with $\boldsymbol{\gamma=-1.4.}$ Plot B: Weight distribution for the network. The distribution follows a power law behaviour with $\boldsymbol{\gamma=-2.5.}$Plot C: Average strength as a function of degree. Plot D: Average weight as function of the product of the degrees of to connected nodes.

# Users’ interest network

In Figure B A,B we show that the degree and the weight distributions of the users’ interest network follow extremely skewed distributions; in particular the degrees are distributed according to a power law with exponential cutoff and the weights to a power law with a very steep slope. This last information means that weights heterogeneity is not so strong and therefore the weighted structure is not so relevant for understanding the topology.

In Figure B C,D we show the strength as a function of the users’ degree and the dependence of the weight $w_{ij}$on the degrees of the end points $k_{i}$and $k_{j}$. The analysis shows that the strength is proportional to the degree of the users (linear growth). Regarding the dependence of the weight our results show a different beahviour from previous technological networks. While in these cases weight scales with product in a sub-linear way [1], in our case the weight is independent of the degree for a large fraction of the range and increases in a super linear way in the range of professionals. This indicates that stronger links are shared by professional users.

Figure B. Plot A: Degree distribution of the network. The distribution follows a power law behaviour with $\boldsymbol{\gamma=-1.}$ Plot B: Weight distribution for the network. The distribution follows a power law behaviour with $\boldsymbol{\gamma=-5.1.}$Plot C: Average strength as a function of degree. Plot D: Average weight as function of the product of the degrees of to connected nodes.

In Figure C we show the relation between users' degree and their activity in the debate in terms of number of hashtags. Low degree nodes show a sub-linear growth of the activity, while high degree nodes show a super-linear behavior. A user has low degree because it has been poorly involved in the debate (it used a small number of hashtag) or because it used many not popular hashtags. High degree nodes ('professionals') have been involved in many different topic (more or less popular).

Figure C. Users’ activities as a function of their degree. The small blue points represent the scattered values. The large cyan points the average values for each degree.

# Additional temporal features

It is trivial to observe that in the semantic network, hubs have higher permanence, (they exist for almost the entire time span of the data collection) and that less important nodes just appear for a short period after which they are forgotten (Figure D A). We notice that in the semantic network the permanence time increases linearly and at the same rate for all the nodes. On the other hand, the permanence time for the users’ interest network nodes grows logarithmically with the degree and not linearly (Figure D B). In the users’ network, the permanence time for 'amateurs', almost constant and short, is strongly different from 'professionals' one, that actually increases as the activity of the user increases.

Figure D. Permanence time as a function of node degree. Plot A semantic network. Plot B users’ interest network.

# The 15M case

In this paragraph we present a partial comparison with a second case study based on the Spanish #15M movement. We present this analysis in the supplementary information since a full straightforward comparison is not possible mostly due to the different procedures for collecting the data.

Twitter data have been collected for the period between 25^th^ April to 25^th^ May 2011 and contain activity for almost 87K users who have posted a message containing at lest one of 70 #hashtags relative to the protest. The dataset contains a timestamp and a list of followers for each user. For each user who sent a protest message, the authors of[2] apply a snowball sampling procedure.

In Figure E we show the comparison between the OWS and the 15M movements for the basic topological properties (the ones presented in Figure 2 of the main text) for the semantic network. As we can observe the strength distributions follow a power law with very similar exponents. Also the degree mixing properties are comparable, both indicating a typical disassortative behavior. Interestingly the rich club index shows an opposite tendency between the two movements. For the 15M case the semantic hubs have very few connections among them compared to a randomized case. This is partially due to the data collection method for the Spanish case, where the tweets concerning the most important hashtags have been collected through different queries. Whilst the Occupy movement that emerged in a completely spontaneous way, the Spanish movement has its basis on different pre-existing social protests that merged together in the 15M demonstrations. This initial pre-existing structure could explain the separate use of different hashtags in different communities.

Figure E. Comparison between the 15M dataset (yellow) and OWS movement one (empty points). A: Strength distribution; B Degree correlation; C Rich club index. The fitting lines are displayed only for the 15M movement; for the OWS see main paper.

The users’ interest network is extremely dense and therefore it is computationally expensive in terms of memory. Moreover, the anonymity of the users in the dataset does not allow us to check the hypothesis we tested in the OWS case concerning the relationships between the topological properties and the social role. In any case, in Figure F, we present the comparison between the strength distributions for the two case studies showing that, also for the 15M, the strengths are distributed according to a power law distribution with an exponential cutoff.

Figure F. Strength distribution: comparison between the dataset 15M (in yellow) and the dataset OWS (empty points). The fitting line is displayed only for the 15M movement; the one for OWS is presented in the main paper.

# REFERENCES

1. Barrat A, Barthelemy M, Pastor-Satorras R, Vespignani A (2003) The architecture of complex weighted networks. Proc Natl Acad Sci USA 101: 3747.

2. González-Bailón S, Borge-Holthoefer J, Rivero A, Moreno Y (2011) The dynamics of protest recruitment through an online network. Scientific reports 1: 197.
